# Relationship between subjective well-being and depressive disorders: Novel findings of cohort variations and demographic heterogeneities

**DOI:** 10.3389/fpsyg.2022.1022643

**Published:** 2023-01-10

**Authors:** Chao Li, Yuxin Xia, Yuhan Zhang

**Affiliations:** ^1^Business School, Shandong University, Weihai, China; ^2^HSBC Business School, Peking University, Shenzhen, China

**Keywords:** subjective well-being, happiness, depressive disorders, cohort variations, heterogeneities

## Abstract

This paper uses a large-scale nationally representative dataset, the Chinese General Social Survey, to examine the relationship between subjective well-being and depressive disorders. Statistical results indicate that higher levels of subjective well-being help decrease perceived depression. Robustness checks are carried out using different types of explanatory and dependent variables, various regression models, penalized machine learning methods, instrumental variable approaches, and placebo tests, all of which lend further credence to the above findings. Based on it, heterogeneities in the relationship between subjective well-being and self-rated mental disorders are explored. In respect of variations in age cohorts, it is found that the absolute values of happiness’s estimated coefficients are smaller in the 20–30 and 30–40 age groups, while that in the 40–50 age group increase substantially. In older cohorts, the estimates remain at higher levels while fluctuating to some degree. Furthermore, the significantly negative interaction between happiness and age proves that age amplifies subjective well-being’s effect on perceived depressive disorders. With age increasing, the impact of happiness on reducing perceived depression tends to be stronger. Therefore, for older people, subjective well-being plays a more important role in suppressing self-rated depression. Heterogeneities of the relationship between happiness and perceived depressive disorders in subgroups with different demographic characteristics are also investigated. It is found that the negative correlation between subjective well-being and self-rated depression is stronger among those with higher educational levels, living in urban areas, being members of the Communist Party of China, having pensions, and owning more housing assets. However, gender, ethnic identity, religious belief, and marital status exert no significant moderating effects.

## 1. Introduction

Subjective well-being is widely used in the area of positive psychology and Diener et al. (1999) give its classical definition from a hedonistic perspective. They believe that subjective well-being includes both cognitive assessment (i.e., overall life satisfaction) and affective well-being (comprising positive and negative emotions). This concept is further developed by introducing eudaimonic well-being (Ryan and Deci, 2001; Deci and Ryan, 2006). Existing literature has studied different factors related to subjective well-being from multiple aspects (Kor et al., 2019; Arslan and Yıldırım, 2021; Chang and Hsu, 2022; Yan et al., 2022; Yang et al., 2022). Among them, the relationship between subjective well-being and health is one of the most important areas. Studies have shown that many indicators of health status, such as the frequency of visiting doctors and hospitalizations, the risk of chronic disease, and use of healthcare, are associated with subjective well-being (Goel et al., 2018; Rosella et al., 2018; De Prophetis et al., 2020).

Psychological research has focused on the relationship between subjective well-being and mental health, although earlier studies do not clearly define the boundary between the two factors. Based on the classic definition of subjective well-being using its key indicators, life satisfaction, and happiness, the relationship between subjective well-being and mental health is investigated (Keyes, 2006; Cavioni et al., 2021). It is found that life satisfaction and happiness are more strongly associated with mental health than other factors such as income, socio-economic status, physical health, human capital, working conditions, family background, and so on (Lombardo et al., 2018). Moreover, happiness is an important predictor of mental health, and people with lower happiness levels use mental health services more frequently (Michalski et al., 2022). In terms of mental disorders, which is another side of mental condition (Defeyter et al., 2021), some scholars believe that anxiety, loneliness, and psychological stress are also closely related to subjective well-being (Ventura-León et al., 2022). In addition, psychological capital, which is an important aspect of mental health, is also found to be positively correlated with happiness (Datu and Valdez, 2019; Turliuc and Candel, 2021; Shan et al., 2022). Based on this, hypothesis 1 can be proposed.

*Hypothesis1*: Subjective well-being is negatively related with depressive disorders.

Furthermore, existing studies also consider the relationship between subjective well-being and mental health in different age cohorts and find heterogeneities. For example, it is demonstrated that the subjective well-being of older adults is higher correlated with mental health (Kim and Sok, 2013; Won et al., 2021). Research on depression in children has shown that those in families with less conflict, higher levels of parental support and more communication have higher levels of happiness, making them mentally healthier (Lee et al., 2006; Morris et al., 2007). Therefore, happiness is one of the important indicators to measure the mental welfare of children. Besides, studies on college students reveal that stressful life experiences reduce their subjective well-being, which may lead to a higher rate of mental illness and even suicidal tendencies (Liu et al., 2022). In addition, previous studies have shown that there are variations in mental health status across groups with different demographic characteristics. Since people’s education level can have an impact on their socio-economic status and availability of medical services, compared to those with lower education levels, respondents who have been educated to a relatively high level have more happiness and less self-reported depressive disorders (Lai et al., 2020; Taple et al., 2020; Chrzastek et al., 2021) In terms of gender, previous studies have shown that women have poorer mental health and are more likely to be depressed and anxious (Zhao et al., 2020; Smith and Mazure, 2021). Besides, since married couples share each other’s social, economic, and psychological resources (Lindström and Rosvall, 2012), marriage is significantly associated with a reduction in people’s perceived anxiety and depression (Le et al., 2020). Based on this, hypothesis 2 can be proposed.

*Hypothesis2*: Cohort and demographic characteristics moderate the relationship between subjective well-being and depressive disorders.

Although existing research has considered the relationship between subjective well-being and mental health, whether subjective well-being is significantly negatively correlated with depressed disorders and whether the relationship is statistically robust needed to be empirically and systematically tested. More importantly, some studies focusing on specific groups have shown variations in the relationship, but results using different samples are not comparable. Therefore, whether there are cohorts and demographic heterogeneities in the relationship between subjective well-being and depressive disorders remains to be specifically examined. Based on this, this paper systematically analyzes the relationship between subjective well-being and perceived depression by using a high-quality, large-scale, comprehensive, and nationally representative microdata in China, the Chinese General Social Survey. We also conduct robustness tests of the relationship from multiple perspectives, including using different explanatory and dependent variables, various regression models, penalized machine learning methods, instrumental variable approaches, and placebo tests. Furthermore, a detailed heterogeneity analysis of the relationship between subjective well-being and self-rated depressive disorders is carried out. The research framework is illustrated as follows:
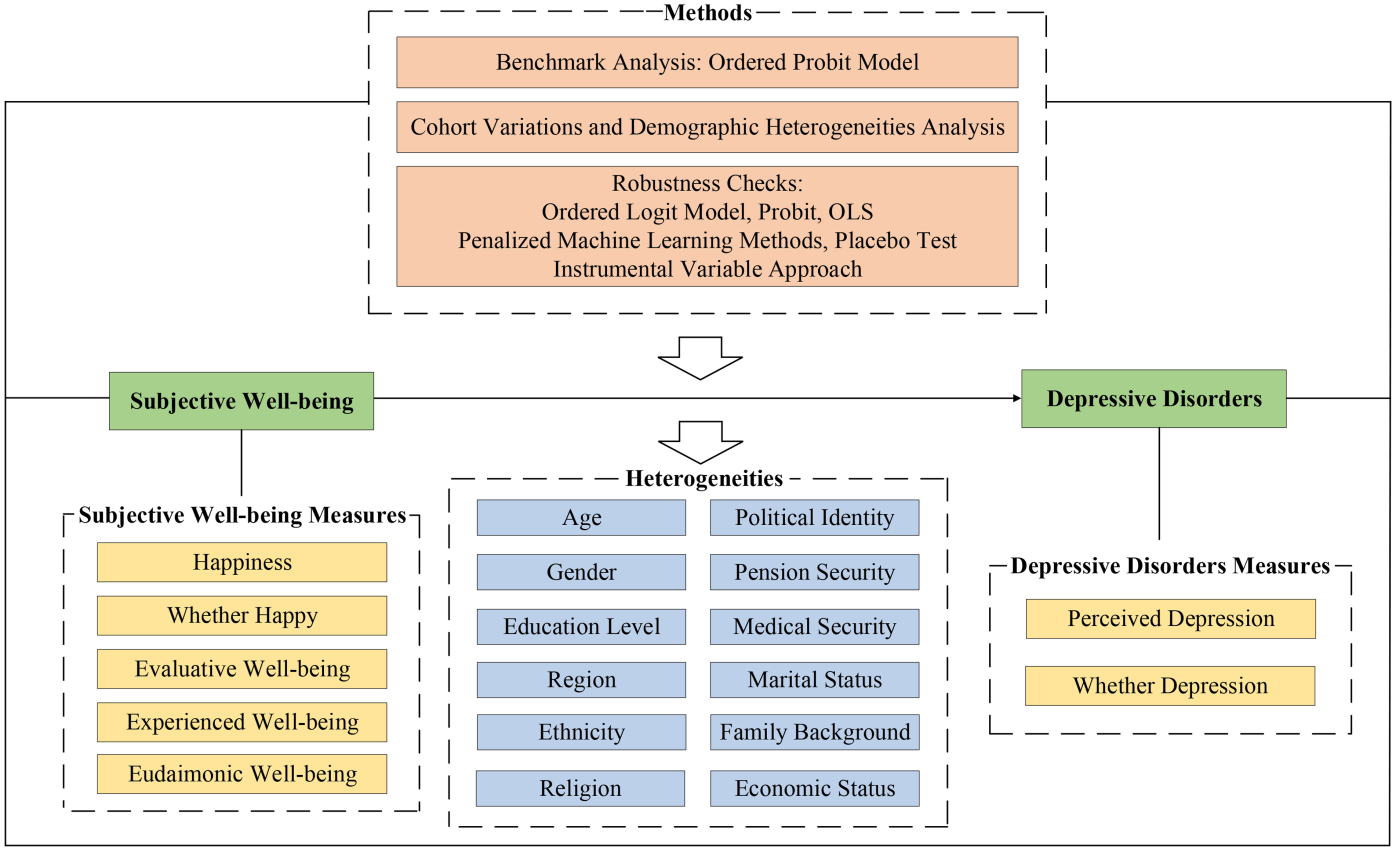


## 2. Materials, measures, and methods

### 2.1. Data source

The dataset used in the paper is the Chinese General Social Survey (CGSS) from 2017 to 2018, which is one of the largest scale and most important micro datasets in China. The sample sizes for the 2017 and 2018 waves are 12,534 and 12,744, respectively, to represent the adult population of Chinese Mainland (excluding Hong Kong, Macao, Taiwan, and Tibet) over 18 years old, which is the universe of CGSS. CGSS aims to systematically and comprehensively reflect the characteristics and trends of Chinese society. There are mainly two reasons for using CGSS. First, CGSS directly surveys people’s overall subjective well-being, the happiness level. Meanwhile, other well-being indicators including the subjective evaluation of well-being (life satisfaction), experienced well-being (the impressions that people experience in their lives), and eudaimonic well-being (sense of purpose and meaning in life) are also measured in an additional module of CGSS. Second, CGSS asks respondents about the degree of their depressive disorders and comprehensively contains influencing factors of perceived depression. Therefore, it is very suitable to conduct research on depressive disorders using CGSS. The data that support the findings of this study are available from the Chinese General Social Survey (CGSS).^1^

### 2.2. Measures

Happiness is the dependent variable and perceived depression is the independent one in this study. The main dependent variable in this paper is the level of perceived depressive disorders, coming from the question in the core module of CGSS: “To what extent do you feel depressed?.” Answers to this question are based on the Likert scale from 1 to 5, including “1-not depressed,” “2-mildly depressed,” “3-moderately depressed,” “4-very depressed,” and “5-severely depressed,” respectively. The explanatory variable is the overall degree of happiness, directly measuring people’s subjective well-being. This variable comes from respondents’ answer to “In general, do you feel your life is happy?.” This question divides happiness levels from 1 to 5 into very unhappy, relatively unhappy, cannot say happy or unhappy, relatively happy, and very happy. The above measures of the dependent and explanatory variables are widely used in the existing literature, as shown in the supplementary introduction to the literature using the key variables in the Supplementary materials. Based on the literature concerning influencing factors of depressive disorders (e.g., Rosenquist et al., 2011; Ustun, 2021), in order to avoid omitted variables bias to the greatest extent, we control six types of variables, including demographic characteristics, human capital characteristics, social characteristics, working characteristics, family characteristics, and regional and time-fixed effects. (1) Demographic characteristics include age, the square of age, and gender. (2) Human capital characteristics include education level and whether the respondent is a migrant. (3) Social characteristics include whether the respondent’s Hukou^2^ is urban, whether she/he belongs to ethnic minorities, whether she/he is a religious believer and Communist Party of China (CPC) member, and social contact frequency. (4) Working characteristics include personal income and whether the respondent has pension and medical insurance. (5) Family characteristics include whether the respondent is married, her/his number of children, family size, and the number of housing assets. (6) Regional and time characteristics include provincial and time dummies. The descriptive statistics of the above variables are shown in Supplementary Table 1.

**Figure 1:**
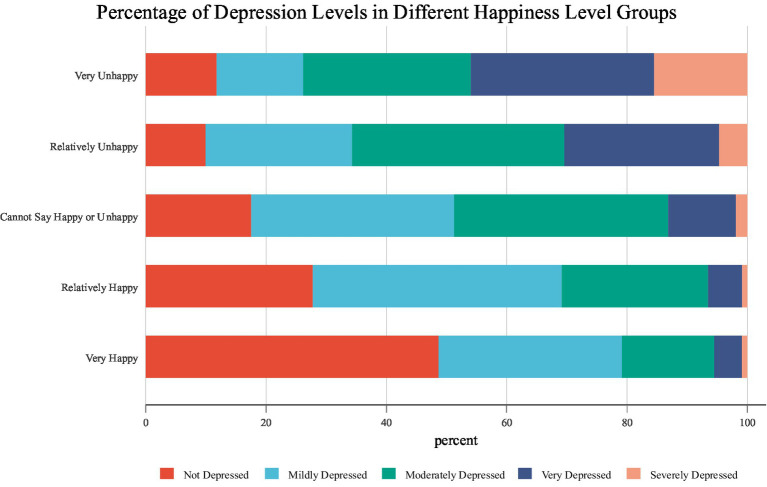
Relationship between subjective well-being and perceived depressive disorders.

Preliminary statistical results of the relationship between happiness and self-rated depressive disorders are illustrated in Figure 1. It is shown that in the group reporting “very unhappy,” the proportion of those who are not depressed is only 11.78%. At the same time, very depressed and severely depressed respondents account for nearly half in total, which are 30.46 and 15.52%, respectively. In groups changing from very unhappy to very happy, the proportion of those with higher levels of depression decreases significantly. In the very happy group, the percentage of not depressed increases nearly five times to 48.67%, compared with that in the very unhappy group. Meanwhile, the proportions of the very depressed and severely depressed drop to 4.59 and 0.92%, respectively. The above descriptive statistical results preliminarily reveal that happiness seems to be negatively correlated with depression, and the higher the happiness level, the less severe the depressive disorders. The relationship between the two factors is further rigorously tested in the following sections.

### 2.3. Methods

Because the dependent variable, perceived depressive disorders, is an ordered variable, the Ordered Probit model (Oprobit) is applied in the benchmark regressions. Specifically, the model is constructed by dividing the sample into five groups according to their depression levels. Groups g = 1 to 5 represent those who are not depressed, mildly depressed, moderately depressed, very depressed, and severely depressed, respectively. In the Ordered Probit model, the probability of a given observation i in group g is denoted as $p_{gi}$ and


$$\begin{array}{l} p_{gi}=\mathrm{Pr}\left(Perceived\_Depression_{i}=g\right)\\ \;\;\;\;\;\;\;\;\;=\mathrm{Pr}\left(\kappa_{g-1}<\alpha +\beta Happiness_{i}+x_{i}^{\prime }\gamma +\varepsilon_{i}\leq \kappa_{g}\right)\\ \;\;\;\;\;\;\;\;\;=\phi \left(\kappa_{g}-\alpha -\beta Happiness_{i}-x_{i}^{\prime }\gamma \right)\\ \;\;\;\;\;\;\;\;\;-\phi \left(\kappa_{g-1}-\alpha -\beta Happiness_{i}-x_{i}^{\prime }\gamma \right) \end{array}$$

where Perceived_Depression and Happiness are the dependent and explanatory variables, $x_{i}^{\prime }$ is a vector of control variables described above, $\kappa_{0}$ is defined as −∞ , $\kappa_{5}$ is defined as +∞, and Φ(⋅) is the standard normal cumulative distribution function. Therefore, the log likelihood of the maximum likelihood estimation (MLE) is


$$lnL=\overset{N}{\underset{i=1}{\sum }}\overset{5}{\underset{g=1}{\sum }}I_{g}\left(Perceived\_Depression_{i}\right)\ln p_{gi}$$

where $\begin{array}{l} I_{g}\left(Perceived\_Depression_{i}\right)=\\ \;\;\;\;\;\;\{\begin{array}{c} 1,ifPerceived\_Depression_{i}=g\\ 0,if\;Perceived\_Depression_{i}\neq g \end{array} \end{array}$ and N is the sample size. Based on this, β and γ are estimated by $\underset{\beta ,\gamma \;}{\max}lnL$ .

## 3. Results

### 3.1. Benchmark results

Estimation results of above Ordered Probit model are shown in Table 1. Column (1) is the regression result without including any control variables, showing that happiness is significantly and negatively correlated with perceived depressive disorders. In columns (2)–(7), we gradually add demographic characteristics, human capital characteristics, social characteristics, working characteristics, family background, and regional and time-fixed effects in regressions. It is demonstrated that the estimated coefficients of subjective well-being are all significantly negative at the 1% level in all regressions, suggesting that the higher the level of happiness, the lower the self-rated depressive disorders. In addition, with gradually including control variables in different dimensions, the estimates of happiness decrease slightly but keep relatively stable. This reflects that the significant relationship between happiness and self-rated depressive disorders is not affected by other variables and very robust. Besides, estimated results of the control variables are basically in line with theoretical expectations. First of all, gender is an important factor in affecting the perceived depressive disorders. This paper shows that women’s self-rated depression is higher, which is consistent with the existing literature. For example, Zhao et al. (2020) and Shafer (2021) also find that females are more likely to be depressed, due to the fact that women typically take on the majority of household chores and childcare responsibilities. In terms of human capital, the results of this paper demonstrate that education level is negatively correlated with depression. This may be attributed to the fact that highly educated individuals have higher socio-economic status and better living conditions, and thus fewer mental disorders (Lai et al., 2020; Taple et al., 2020; Chrzastek et al., 2021). However, due to multi-collinearity problem, the estimated coefficient on education level is not significant when other characteristics are controlled. In respect of working characteristics, those with lower-income levels have more mental disorders (Ridley et al., 2020; Zare et al., 2022). In addition, we find that self-rated depression is higher among people with religious beliefs. This is consistent with existing findings that in a more and more secularized society, religious believers are likely to bear more pressure (Upenieks, 2022). Besides, results also show that political identity is an important factor affecting self-rated depression. In China, being a member of Community Party of China (CPC) means having a higher level of social status (Chapman et al., 2020; LaMontagne et al., 2020), so they are less likely to suffer from high depression as demonstrated in this paper’s results. In addition, it has been suggested that because married couples share each other’s social, economic, and psychological resources, marriage is significantly associated with a reduction in people’s perceived anxiety and depression (Lindström and Rosvall, 2012). This study also supports this relationship, indicating that married people have lower levels of perceived depression. In addition, we find that the number of children is positively associated with self-rated depression. This is mainly due to the fact that raising children requires large investment of time and financial resources, which makes people with more children are more likely to suffer from depression (Hentges et al., 2020; Giannelis et al., 2021).

**Table 1:** Relationship between subjective well-being and perceived depressive disorders.

Model	(1) Oprobit	(2) Oprobit	(3) Oprobit	(4) Oprobit	(5) Oprobit	(6) Oprobit	(7) Oprobit
Variable	Perceived depression	Perceived depression	Perceived depression	Perceived depression	Perceived depression	Perceived depression	Perceived depression
Happiness	−0.415^***^ (0.009)	−0.421^***^ (0.009)	−0.406^***^ (0.009)	−0.400^***^ (0.009)	−0.396^***^ (0.010)	−0.390^***^ (0.010)	−0.386^***^ (0.010)
Whether female		0.162^***^ (0.014)	0.135^***^ (0.014)	0.136^***^ (0.014)	0.118^***^ (0.015)	0.115^***^ (0.015)	0.135^***^ (0.015)
Age		0.005^**^ (0.002)	−0.005^**^ (0.002)	−0.001 (0.002)	0.003 (0.003)	0.009^***^ (0.003)	0.009^***^ (0.003)
Age_squared		−0.000 (0.000)	0.000^***^ (0.000)	0.000^*^ (0.000)	0.000 (0.000)	−0.000^***^ (0.000)	−0.000^**^ (0.000)
Education level			−0.039^***^ (0.002)	−0.016^***^ (0.003)	−0.012^***^ (0.003)	−0.010^***^ (0.003)	−0.003 (0.003)
Whether migrants			−0.096^***^ (0.021)	−0.108^***^ (0.021)	−0.094^***^ (0.022)	−0.101^***^ (0.023)	0.003 (0.024)
Whether Hukou in urban				−0.210^***^ (0.016)	−0.189^***^ (0.017)	−0.170^***^ (0.017)	−0.096^***^ (0.018)
Whether ethnic minorities				0.119^***^ (0.027)	0.110^***^ (0.028)	0.100^***^ (0.028)	−0.037 (0.033)
Whether religious believer				0.052^**^ (0.023)	0.046^*^ (0.024)	0.039 (0.024)	0.063^**^ (0.025)
Whether CPC member				−0.124^***^ (0.025)	−0.106^***^ (0.026)	−0.093^***^ (0.026)	−0.106^***^ (0.026)
Social contacts frequency				−0.026^***^ (0.007)	−0.026^***^ (0.007)	−0.027^***^ (0.007)	−0.024^***^ (0.007)
ln_Income					−0.018^***^ (0.002)	−0.017^***^ (0.002)	−0.013^***^ (0.002)
Whether having pension					−0.033^*^ (0.018)	−0.033^*^ (0.018)	−0.014 (0.018)
Whether having medical insurance					0.086^***^ (0.030)	0.097^***^ (0.030)	0.082^***^ (0.030)
Whether married						−0.104^***^ (0.019)	−0.095^***^ (0.019)
Number of children						0.050^***^ (0.007)	0.030^***^ (0.007)
Family size						−0.009^*^ (0.005)	−0.010^*^ (0.005)
Number of houses						−0.011 (0.012)	0.003 (0.012)
Year dummies	No	No	No	No	No	No	Yes
Province dummies	No	No	No	No	No	No	Yes
Observations	25278	25278	25195	25111	23749	23515	23515
Pseudo *R*^2^	0.037	0.042	0.046	0.050	0.051	0.052	0.060

***, **, and * indicate significance at the levels of 1, 5, and 10%, respectively. The values in parentheses are standard errors robust to heteroskedasticity. Yes means the corresponding variables are controlled in the regression, while No means not controlled. Happiness is classified from 1 to 5: 1-very unhappy, 2-relatively unhappy, 3-cannot say happy or unhappy, 4-relatively happy, 5-very happy. Perceived depression is classified from 1 to 5: 1-not depressed, 2-mildly depressed, 3-moderately depressed, 4-very depressed, 5-severely depressed.

### 3.2. Cohort variations and demographic heterogeneities

**Figure 2:**
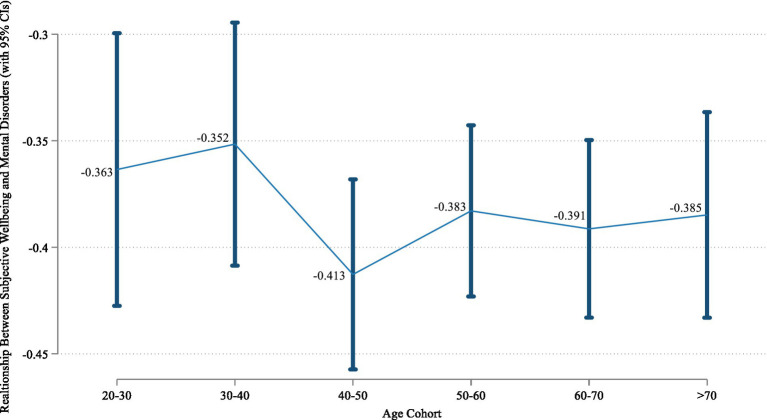
Cohort variations.

In order to investigate the heterogeneous relationship between happiness and perceived depression in different age cohorts, we divide the sample into different age groups and perform subsample regressions. Results of analytical statistics of different age cohorts are demonstrated in Supplementary Tables 2–7. Estimated coefficients and their 95% confidence intervals of different age cohorts are illustrated in Figure 2 and detailed regression results are shown in Supplementary Table 2. It is demonstrated that the relationships between happiness and depression in all subsamples are significantly negative, further proving the robustness of the benchmark regression results. Furthermore, we find that the absolute values of the estimated coefficients are smaller for age groups in the 20–30 and 30–40, while that in the 40–50 age group increase notably. In older cohorts, the estimated coefficients fluctuate to some extent but remain at higher levels. This implies that the negative relationship between happiness and depression tends to be more prominent with age increasing. To further test this conclusion, we analyze whether age moderates the relationship between the two factors, and results are shown in the column (1) of Table 2. It is indicated that the interaction between happiness and age is significantly negative. It confirms the significant moderating effect of age on the happiness’s impact, certifying that the influence of happiness on reducing perceived depression significantly increases with age rising.

**Table 2:** Demographic heterogeneities.

Model	(1) Oprobit	(2) Oprobit	(3) Oprobit	(4) Oprobit	(5) Oprobit	(6) Oprobit
Moderator variable	Age	Whether female	Education level	Whether Hukou in urban	Whether ethnic minorities	Whether religious believer
Interaction between happiness and moderator	−0.001^**^ (0.001)	−0.025 (0.018)	−0.013^***^ (0.003)	−0.079^***^ (0.020)	0.057 (0.035)	−0.322 (0.029)
Happiness	−0.325^***^ (0.032)	−0.373^***^ (0.014)	−0.330^***^ (0.017)	−0.362^***^ (0.011)	−0.391^***^ (0.010)	−0.383^***^ (0.010)
Moderator variable	0.014^***^ (0.004)	0.231^***^ (0.072)	0.048^***^ (0.013)	0.211^***^ (0.080)	−0.256^*^ (0.140)	0.188 (0.114)
Controls	Yes	Yes	Yes	Yes	Yes	Yes
Observations	23515	23515	23515	23515	23515	23515
Pseudo *R*^2^	0.060	0.060	0.060	0.060	0.060	0.060
**Model**	**(7) Oprobit**	**(8) Oprobit**	**(9) Oprobit**	**(10) Oprobit**	**(11) Oprobit**	**(12) Oprobit**
**Moderator Variable**	**Whether CPC member**	**Whether having pension**	**Whether having medical insurance**	**Whether married**	**Family size**	**Number of houses**
Interaction between happiness and moderator	−0.088^**^ (0.036)	−0.052^***^ (0.020)	0.019 (0.032)	0.004 (0.020)	0.023^***^ (0.005)	−0.045^***^ (0.016)
Happiness	−0.380^***^ (0.010)	−0.250^***^ (0.017)	−0.404^***^ (0.031)	−0.390^***^ (0.018)	−0.450^***^ (0.018)	−0.340^***^ (0.019)
Moderator variable	0.253^*^ (0.148)	0.183^**^ (0.077)	0.012 (0.122)	−0.112 (0.079)	−0.100^***^ (0.021)	0.178^***^ (0.064)
Controls	Yes	Yes	Yes	Yes	Yes	Yes
Observations	23515	23515	23515	23515	23515	23515
Pseudo *R*^2^	0.060	0.060	0.060	0.060	0.060	0.060

Other controls include controls other than the moderator variable.

Moreover, this paper further explores heterogeneities of the relationship between subjective well-being and self-rated depressive disorders in groups with different demographic characteristics, as shown in Table 2. Here, we mainly focus on the estimated coefficients of the interactions between moderating variables and happiness. If the estimate of the interaction term is positive, it means that the moderator reduces the negative correlation between happiness and perceived depression. In contrast, the opposite result would mean that the moderation effect enhances the relationship between the two factors. Results in Table 2 demonstrate that the interaction between gender and happiness is not significant, implying that there is no noticeable gender difference in the negative correlation between subjective well-being and self-rated depression. The interaction term between education level and happiness is significantly negative, suggesting that education moderates the relationship between happiness and perceived depressive disorders. The higher the level of education, the stronger the happiness’s effect on reducing mental disorders. In addition, it is also shown that the correlation is stronger if the individual comes from urban areas. This result is consistent with expectations from theoretical analysis. In China, residents in urban areas have a higher living standard and better social security than those in rural areas, and thus the promotion of their subjective well-being is more helpful to reduce self-rated mental disorders. However, regression results indicate that respondents’ ethnic identity and religious belief do not affect the relationship between happiness and perceived depression.

It is found that political identity plays an important role in moderating the association between happiness and self-rated depressive disorders. Specifically, the improved subjective well-being of CPC members is more conducive to weakening self-rated depressive disorders. This may be related to their higher social status. Furthermore, based on similar logic, it turns out that if the respondent has pension, the negative relationship between her/his subjective well-being and perceived mental disorders is stronger. However, moderating effects do not exist in terms of medical insurance or marriage status. We also found that the larger the family size, the weaker the negative correlation between happiness and perceived depression. Besides, the number of housing assets in the family enhances the negative effect of happiness on self-rated depressive disorders. Overall, heterogeneity analysis shows that the negative relationship between subjective well-being and perceived depression is stronger for individuals with higher levels of education, living standards, and socio-economic status, while gender, ethnic identity, religious belief, and marital status seem not to exert moderating effects.

### 3.3. Robustness checks

Robustness of the relationship between subjective well-being and depressive disorders is examined in the following six aspects. First, we use other three kinds of subjective well-being indicators as explanatory variables in regressions, including evaluative well-being (life satisfaction), experienced well-being (the emotions that people experience in their lives), and eudaimonic well-being (sense of purpose and meaning in life). Results in Supplementary Table 3 further demonstrate that there are significantly negative correlations between different types of subjective well-being measures and perceived depression, confirming the robustness of conclusions in this paper. Second, we perform regressions using the dummy variable from “whether feel depressed or not” as the explained variable. Supplementary Table 4 shows that the association between subjective well-being and this variable is also significantly negative and very robust in all the regressions. Third, we use the Ordered Logit and Ordinary Least Squares models to estimate the relationship between happiness and self-rated depressive disorders, respectively. Results in Supplementary Tables 5, 6 show that no matter which regression model is used and no matter which control variables are included, the estimated coefficients of subjective well-being are all significantly negative.

Fourth, we examine the predictive power of subjective well-being for perceived depression using penalized machine learning methods. Specifically, we use the 10-fold and 20-fold cross-validation approaches to obtain the optimal penalties λ for the Lasso, Ridge, and Elastic Net models to conduct penalized estimation. Supplementary Table 7 and Supplementary Figures 1, 2 show that subjective well-being is a key predictor of depression in all of these penalized models. This further proves the robustness of the relationship between happiness and self-rated depressive disorders. Fifth, considering there may exist endogeneity in the relationship between subjective well-being and perceived depression, the instrumental variable method is further used for the estimation. Specifically, following Mihaylov and Tijdens (2019), we use the degree of automation’s impact on the work performed by workers as the instrumental variable, denoted as *Automation*. Research has shown that automation can help improve subjective well-being through cutting down working time (Acemoglu and Restrepo, 2020). Therefore, this instrumental variable satisfies the correlation requirement. In addition, the instrumental variable is determined by exogenous technological changes and has nothing to do with the characteristics of individual workers, thus satisfying the exogeneity condition. As shown in Supplementary Table 8, all the instrumental variable regressions show that the significantly negative relationship between happiness and self-rated depression is not affected by the potential endogeneity. Sixth, we perform a placebo test of the results. Specifically, we randomly reassign the happiness variable in the sample 1,000 times to generate new samples, respectively, and perform regressions in them to obtain estimates of happiness. The mean value of these 1,000 estimated coefficients is close to 0, more than 90% of their *p*-values are greater than 0.1. Supplementary Figure 3 shows that all the estimated coefficients are greater than −0.079, which is in the benchmark regression result. This further rules out omitted random factors’ interference with the relationship between subjective well-being and mental disorders.

## 4. Conclusion

This paper uses the Chinese General Social Survey to empirically examine the relationship between subjective well-being and perceived depressive disorders. Analytical results suggest that happiness significantly weakens people’s self-rated depression. In respect of cohort variations, the absolute values of happiness’s estimated coefficients are much smaller in the 20–30 and 30–40 age groups. The absolute value of the estimate in the 40–50 age group increases noticeably. In older cohorts, the coefficients remain at higher levels while fluctuating to some degree. The significantly negative interaction between happiness and age proves that age exerts a significant moderating effect on the influence of subjective well-being’s impact. With age rising, the effect of happiness on reducing depression is stronger. Therefore, for older people, subjective well-being plays a more prominent role in suppressing depression. In addition, this study finds that the negative correlation between happiness and self-rated depression is stronger among those with higher education levels, living in urban areas, being members of the Communist Party of China, having pension, and owning more housing assets. However, gender, ethnic identity, religious belief, and marital status have no significant moderating effects. In addition, we use different explanatory and explained variables, different regression models, panelized machine learning methods, instrumental variable approaches, and placebo tests to conduct robustness checks, all of which lend further credence to the above conclusions of this paper.

The shortcomings of this paper are mainly reflected in two aspects. First, the Chinese General Social Survey, which is used in this study, is based on subjective answers, and thus both the explanatory and the explained variables in this paper are subjective indicators without validated questionnaires. Although other variables are further used as dependent variables in the robustness checks, confirming the relationship between happiness and perceived depression, the measures are also subjective indicators. Therefore, we look forward to further testing the relationship between the two factors based on validated questionnaires in the future. Secondly, due to data limitations, R2 in the regression models of this article is relatively low, which means variations in the explanatory variables have limited explanatory power on the changes of the explained variable. Therefore, we look forward to further investigating the results of this research on the basis of more ideal dataset in the future.

## 5. Discussion

This study systematically explores the relationship between subjective well-being and perceived depressive disorders. Previous studies have mainly discussed possible associations between happiness and mental disorders in general, but have not yet confirmed whether happiness is significantly and negatively related to depressive disorders. For example, it has been shown that happiness is an important indicator of mental health (Schuster et al., 2020; Soga et al., 2020; Fukuya et al., 2021). Similar to income, socio-economic status, health, human capital, and family background, happiness is also a very important element impacting mental health, and the correlation between the two factors is very strong (Lombardo et al., 2018). Specifically, research based on the Canadian Community Health Survey (CCHS 2005–2014) has found that individuals with lower subjective well-being generally have poorer mental health, and therefore use mental health services more frequently (Michalski et al., 2022) Moreover, depression is also a psychological state that is opposite to happiness. Some studies show that the more anxiety and depression people feel, the lower their subjective well-being (Freire and Ferreira, 2020; Yildirim and Balahmar, 2020; Ventura-León et al., 2022). However, whether happiness can weaken people’s perceived depressive disorders still remains to be empirically tested. Therefore, this paper extends the conclusions on the relationship between happiness and mental health in the above literature, demonstrating that subjective well-being plays an important role in weakening people’s depression. Specifically, this study proves that higher levels of subjective well-being help to decrease perceived depression. This conclusion is robust to different types of explanatory and dependent variables, various regression models, penalized machine learning methods, instrumental variable approaches, and placebo tests.

In addition, we further find age cohort variations in the relationship between subjective well-being and perceived depression. In terms of age cohorts, existing studies have mainly explored the relationship between the two factors for a specific age group, while results using different samples are lack of comparability. For example, research based on the elderly shows that happiness of older adult is highly correlated with their mental health (Won et al., 2021). Children who grow up in families with higher levels of parental support and better atmosphere have higher levels of happiness, making them mentally healthier (Lee et al., 2006; Morris et al., 2007) In addition, studies on college students reveal that stressful life experiences reduce their subjective well-being, which leads to a higher rate of mental illness (Liu et al., 2022). However, data sources, explanatory variables, dependent variables, and statistical methods used in different researches vary greatly, so their analytical results are not comparable. Therefore, in respect of age heterogeneity, a representative large-scale sample comprising all age groups is required for a detailed comparative study on different age cohorts. The CGSS data used in this paper facilitate such analysis. Specifically, this study finds that the absolute values of happiness’s estimated coefficients are much smaller in the 20–30 and 30–40 age groups, while they in the 40–50 age group increase noticeably. With age rising, the effect of happiness on reducing depression is stronger. Therefore, for older people, subjective well-being plays a more prominent role in suppressing depression.

At the same time, compared with existing studies that support the positive relationship between happiness and mental health (Lombardo et al., 2018; Schuster et al., 2020; Soga et al., 2020; Fukuya et al., 2021), this paper further explores heterogeneities of the correlation between the two factors across groups with different demographic characteristics. Heterogeneity analysis shows that the negative correlation between happiness and depression is stronger among those with higher education levels, living in urban areas, being members of the Communist Party of China, having pension, and owning more housing assets. However, gender, ethnic identity, religious belief, and marital status have no significant moderating effects. These findings inspire us to improve the subjective well-being of vulnerable subgroups more specifically and thus to reduce the adverse effects of depression.

In addition, some other results in this research are also worth mentioning. For example, consistent with the existing literature, this paper also finds that gender is an important factor in affecting depressive disorders. Zhao et al. (2020) and Shafer (2021) find that due to gender disparities in depression-related genes and the fact that women typically undertake the majority of household chores, females are more likely to be depressed. It is also found that those with lower-income experience higher levels of depression, which is consistent with previous studies by Ridley et al. (2020) and Zare et al. (2022). Because of the protective effects of social networks on mental health (Werner-Seidler et al., 2017), we find that those participating in more social activities are less likely to suffer from depression. Furthermore, divorced or unmarried groups have poorer mental health, related to findings in the literature that marriage provides shared social, economic, and psychological resources (Lindström and Rosvall, 2012). In addition, parenting stress makes people with more children more likely to suffer from depression (Giannelis et al., 2021), while parents without children are less depressed (Evenson and Simon, 2005). This study also supports this relationship. Moreover, it is discovered that both political identity and religious belief are important factors affecting mental disorders, but only political identity moderates the relationship between subjective well-being and depression, which is not paid attention to in existing literature.

This paper clarifies the relationship between subjective well-being and perceived depressive disorders, which has important clinical implications for improving happiness to alleviate the adverse effects of depression on mental health. Therefore, the results of this study have strong practical value. First, this study empirically confirms that subjective well-being plays a significant role in mitigating people’s depression. This means that in the clinical treatment of depressive disorders, in addition to traditional interventions such as medication and psychotherapy, subjective well-being’s benefits should be emphasized. Measures that have been proved to be helpful to improve people’s happiness can be considered to reduce their mental disorders. Second, this study finds that, in different age cohorts, the absolute values of the estimated coefficients are smaller for age groups in the 20–30 and 30–40, while that in the 40–50 age group increase notably. Specifically, for older people, subjective well-being plays a more prominent role in suppressing depression. The elderly are also more adversely affected by mental disorders. Therefore, we should focus more on their well-being. Specific measures can be considered such as improving the quality of elderly services and pension system, as well as establishing an infrastructure more suitable for the elderly, so as to enhance happiness for older people. All of them will help to reduce the negative impact of depressive disorders on them. Third, policymakers should pay more attention to vulnerable subgroups, focusing on their subjective well-being and mental health. Specifically, we find that the negative correlation between happiness and depression is stronger among those with higher education levels, living in urban areas, being members of the Communist Party of China, having pension, and owning more housing assets. Therefore, for individuals with lower educational levels, living in rural areas, with lower socio-economic status, and uncovered by social security, their living standards and subjective well-being should be emphasized, so as to weaken the negative impact of depressive disorders.

## Data availability statement

The original contributions presented in the study are included in the article/Supplementary material, further inquiries can be directed to the corresponding author.

## Ethics statement

The studies involving human participants were reviewed and approved by Institutional Review Board, Renmin University of China. The patients/participants provided their written informed consent to participate in this study.

## Author contributions

CL contributed to the conception and design of the study and performed the statistical analysis and wrote the first draft of the manuscript. YX and YZ generated the tables and figures, respectively, based on CL’s analysis. YX worked on revisions of the manuscript. All authors provided critical feedback and approved the final submission.

## Funding

This work was supported by the Project of Natural Science Foundation of China (grant number 71973081); Humanities and Social Science Research Project of the Ministry of Education of China (grant number 19YJC790055); the Project of Natural Science Foundation of Shandong Province, China (grant number ZR2020QG038); the Project of Social Science Foundation of Shandong Province, China (grant number 19DJJJ08); and the Project of Teaching Reform of Shandong University, Weihai (grant number Y2022007).

## Conflict of interest

The authors declare that the research was conducted in the absence of any commercial or financial relationships that could be construed as a potential conflict of interest.

## Publisher’s note

All claims expressed in this article are solely those of the authors and do not necessarily represent those of their affiliated organizations, or those of the publisher, the editors and the reviewers. Any product that may be evaluated in this article, or claim that may be made by its manufacturer, is not guaranteed or endorsed by the publisher.

## Supplementary material

The Supplementary material for this article can be found online at: https://www.frontiersin.org/articles/10.3389/fpsyg.2022.1022643/full#supplementary-material

## Footnotes

^1^ http://cgss.ruc.edu.cn/English/Home.htm

^2^Hukou is a system of household registration used in mainland China, mainly identifying a person as a rural or urban resident.
