# Supplementary material for: Relationship between subjective well-being and depressive disorders: Novel findings of cohort variations and demographic heterogeneities
Source: Front Psychol. 2023 Jan 10;13:1022643. doi: 10.3389/fpsyg.2022.1022643 (PMC9872016; doi:10.3389/fpsyg.2022.1022643)
Supplement: Supplementary file 1 [file Data_Sheet_1.docx]

Supplementary Material

**Supplementary Table 1.** Summary statistics.

| **Variable** | **Description** | **Obs** | **Mean** | **Std. Dev.** | **Min** | **Max** |
| --- | --- | --- | --- | --- | --- | --- |
| Explanatory Variables |  |  |  |  |  |  |
| Happiness | 1-5 levels | 25333 | 3.877 | 0.834 | 1 | 5 |
| Whether happy | Yes=1, No=0 | 25333 | 0.786 | 0.410 | 0 | 1 |
| Dependent Variable |  |  |  |  |  |  |
| Perceived depression | 1-5 levels | 25312 | 2.162 | 0.982 | 1 | 5 |
| Control Variables |  |  |  |  |  |  |
| Demographic Characteristics |  |  |  |  |  |  |
| Whether female | Yes=1, No=0 | 25369 | 0.531 | 0.499 | 0 | 1 |
| Age | Age | 25369 | 51.423 | 16.911 | 18 | 118 |
| Age_squared | Squared term of age | 25369 | 2930.297 | 1760.003 | 324 | 13924 |
| Human Capital Characteristics |  |  |  |  |  |  |
| Education level | 1-13 levels | 25355 | 5.109 | 3.282 | 1 | 13 |
| Whether migrants | Yes=1, No=0 | 25298 | 0.139 | 0.346 | 0 | 1 |
| Social Characteristics |  |  |  |  |  |  |
| Whether Hukou in urban | Yes=1, No=0 | 25295 | 0.373 | 0.484 | 0 | 1 |
| Whether ethnic minorities | Yes=1, No=0 | 25369 | 0.074 | 0.262 | 0 | 1 |
| Whether religious believer | Yes=1, No=0 | 25369 | 0.105 | 0.307 | 0 | 1 |
| Whether CPC member | Yes=1, No=0 | 25336 | 0.111 | 0.315 | 0 | 1 |
| Social contacts frequency | 1-5 levels | 25362 | 2.722 | 1.058 | 1 | 5 |
| Working Characteristics |  |  |  |  |  |  |
| ln_Income | Logarithm of personal total income (RMB) | 24008 | 8.363 | 3.862 | 0 | 16.117 |
| Whether having pension | Yes=1, No=0 | 25308 | 0.740 | 0.439 | 0 | 1 |
| Whether having medical insurance | Yes=1, No=0 | 25333 | 0.928 | 0.258 | 0 | 1 |
| Family Characteristics |  |  |  |  |  |  |
| Whether married | Yes=1, No=0 | 25369 | 0.752 | 0.432 | 0 | 1 |
| Number of children | Number of children in the family | 25320 | 1.698 | 1.312 | 0 | 32 |
| Family size | Number of members in the family | 25336 | 2.812 | 1.492 | 1 | 44 |
| Number of houses | Number of houses in the family | 25086 | 1.099 | 0.653 | 0 | 15 |
| Year dummies |  |  |  |  |  |  |
| Province dummies |  |  |  |  |  |  |

Note: Happiness is classified from 1 to 5: 1-very unhappy, 2-relatively unhappy, 3-cannot say happy or unhappy, 4-relatively happy, 5-very happy. Perceived depression is classified from 1 to 5: 1-not depressed, 2-mildly depressed, 3-moderately depressed, 4-very depressed, 5-severely depressed. The education level is classified from 1 to 13: 1-without any education, 2-kindergarten, 3-primary school, 4-junior high school, 5-vocational high school, 6-ordinary high school, 7-techinical secondary school, 8-technical high school, 9-junior college (adult education), 10-junior college (regular education), 11-undergraduate (adult education), 12-undergraduate (regular education), 13-postgraduate and above. The social contacts frequency is classified from 1 to 5: 1-never, 2-seldom, 3-sometimes, 4-often, 5-always. The meanings of dummy variables are all “Yes=1, No=0”. Hukou is a system of household registration used in mainland China, mainly identifying a person as a rural or urban resident. CPC is Communist Party of China.

**Supplementary Table 2.** Summary statistics of people in the age of 20-30

| **Variable** | **Description** | **Obs** | **Mean** | **Std. Dev.** | **Min** | **Max** |
| --- | --- | --- | --- | --- | --- | --- |
| Explanatory Variables |  |  |  |  |  |  |
| Happiness | 1-5 levels | 2981 | 3.911 | 0.756 | 1 | 5 |
| Dependent Variable |  |  |  |  |  |  |
| Perceived Depression | 1-5 levels | 2981 | 2.065 | 0.899 | 1 | 5 |
| Control Variables |  |  |  |  |  |  |
| Demographic Characteristics |  |  |  |  |  |  |
| Whether female | Yes=1, No=0 | 2981 | 0.528 | 0.499 | 0 | 1 |
| Age | Age | 2981 | 26.030 | 2.861 | 21 | 30 |
| Age_squared | Squared term of age | 2981 | 685.755 | 147.485 | 441 | 900 |
| Human Capital Characteristics |  |  |  |  |  |  |
| Education level | 1-13 levels | 2979 | 8.218 | 3.500 | 1 | 13 |
| Whether migrants | Yes=1, No=0 | 2961 | 0.316 | 0.465 | 0 | 1 |
| Social Characteristics |  |  |  |  |  |  |
| Whether Hukou in urban | Yes=1, No=0 | 2967 | 0.381 | 0.486 | 0 | 1 |
| Whether ethnic minorities | Yes=1, No=0 | 2981 | 0.075 | 0.264 | 0 | 1 |
| Whether religious believer | Yes=1, No=0 | 2981 | 0.082 | 0.274 | 0 | 1 |
| Whether CPC member | Yes=1, No=0 | 2967 | 0.080 | 0.272 | 0 | 1 |
| Social contacts frequency | 1-5 levels | 2981 | 2.770 | 0.967 | 1 | 5 |
| Working Characteristics |  |  |  |  |  |  |
| ln_Income | Logarithm of personal total income (RMB) | 2770 | 8.467 | 4.379 | 0 | 16.113 |
| Whether having pension | Yes=1, No=0 | 2963 | 0.547 | 0.498 | 0 | 1 |
| Whether having medical insurance | Yes=1, No=0 | 2974 | 0.900 | 0.300 | 0 | 1 |
| Family Characteristics |  |  |  |  |  |  |
| Whether married | Yes=1, No=0 | 2981 | 0.470 | 0.499 | 0 | 1 |
| Number of children | Number of children in the family | 2979 | 0.564 | 0.823 | 0 | 12 |
| Family size | Number of members in the family | 2976 | 2.997 | 1.758 | 1 | 44 |
| Number of houses | Number of houses in the family | 2927 | 1.104 | 0.858 | 0 | 15 |
| Year dummies |  |  |  |  |  |  |
| Province dummies |  |  |  |  |  |  |

**Supplementary Table 3.** Summary statistics of people in the age of 30-40

| **Variable** | **Description** | **Obs.** | **Mean** | **Std. Dev.** | **Min** | **Max** |
| --- | --- | --- | --- | --- | --- | --- |
| Explanatory Variables |  |  |  |  |  |  |
| Happiness | 1-5 levels | 3669 | 3.898 | 0.780 | 1 | 5 |
| Dependent Variable |  |  |  |  |  |  |
| Perceived Depression | 1-5 levels | 3669 | 2.060 | 0.780 | 1 | 5 |
| Control Variables |  |  |  |  |  |  |
| Demographic Characteristics |  |  |  |  |  |  |
| Whether female | Yes=1, No=0 | 3669 | 0.537 | 0.499 | 0 | 1 |
| Age | Age | 3669 | 35.687 | 2.917 | 31 | 40 |
| Age_squared | Squared term of age | 3669 | 1282.055 | 207.854 | 961 | 1600 |
| Human Capital Characteristics |  |  |  |  |  |  |
| Education level | 1-13 levels | 3668 | 6.659 | 3.489 | 1 | 13 |
| Whether migrants | Yes=1, No=0 | 3650 | 0.219 | 0.413 | 0 | 1 |
| Social Characteristics |  |  |  |  |  |  |
| Whether Hukou in urban | Yes=1, No=0 | 3658 | 0.399 | 0.490 | 0 | 1 |
| Whether ethnic minorities | Yes=1, No=0 | 3669 | 0.081 | 0.273 | 0 | 1 |
| Whether religious believer | Yes=1, No=0 | 3669 | 0.095 | 0.293 | 0 | 1 |
| Whether CPC member | Yes=1, No=0 | 3665 | 0.107 | 0.309 | 0 | 1 |
| Social contacts frequency | 1-5 levels | 3669 | 2.746 | 0.941 | 1 | 5 |
| Working Characteristics |  |  |  |  |  |  |
| ln_Income | Logarithm of personal total income (RMB) | 3449 | 9.406 | 3.558 | 0 | 16.107 |
| Whether having pension | Yes=1, No=0 | 3665 | 0.703 | 0.457 | 0 | 1 |
| Whether having medical insurance | Yes=1, No=0 | 3665 | 0.935 | 0.247 | 0 | 1 |
| Family Characteristics |  |  |  |  |  |  |
| Whether married | Yes=1, No=0 | 3669 | 0.873 | 0.333 | 0 | 1 |
| Number of children | Number of children in the family | 3656 | 1.375 | 0.914 | 0 | 20 |
| Family size | Number of members in the family | 3662 | 3.431 | 1.418 | 1 | 12 |
| Number of houses | Number of houses in the family | 3622 | 1.090 | 0.671 | 0 | 9 |
| Year dummies |  |  |  |  |  |  |
| Province dummies |  |  |  |  |  |  |

**Supplementary Table 4.** Summary statistics of people in the age of 40-50

| **Variable** | **Description** | **Obs.** | **Mean** | **Std. Dev.** | **Min** | **Max** |
| --- | --- | --- | --- | --- | --- | --- |
| Explanatory Variables |  |  |  |  |  |  |
| Happiness | 1-5 levels | 4655 | 3.794 | 0.837 | 1 | 5 |
| Dependent Variable |  |  |  |  |  |  |
| Perceived Depression | 1-5 levels | 4655 | 2.163 | 0.972 | 1 | 5 |
| Control Variables |  |  |  |  |  |  |
| Demographic Characteristics |  |  |  |  |  |  |
| Whether female | Yes=1, No=0 | 4655 | 0.545 | 0.498 | 0 | 1 |
| Age | Age | 4655 | 45.948 | 2.810 | 41 | 50 |
| Age_squared | Squared term of age | 4655 | 2119.092 | 256.818 | 1681 | 2500 |
| Human Capital Characteristics |  |  |  |  |  |  |
| Education level | 1-13 levels | 4651 | 4.913 | 2.904 | 1 | 13 |
| Whether migrants | Yes=1, No=0 | 4639 | 0.130 | 0.336 | 0 | 1 |
| Social Characteristics |  |  |  |  |  |  |
| Whether Hukou in urban | Yes=1, No=0 | 4644 | 0.328 | 0.469 | 0 | 1 |
| Whether ethnic minorities | Yes=1, No=0 | 4655 | 0.081 | 0.273 | 0 | 1 |
| Whether religious believer | Yes=1, No=0 | 4655 | 0.096 | 0295 | 0 | 1 |
| Whether CPC member | Yes=1, No=0 | 4652 | 0.086 | 0.281 | 0 | 1 |
| Social contacts frequency | 1-5 levels | 4653 | 2.725 | 1.009 | 1 | 5 |
| Working Characteristics |  |  |  |  |  |  |
| ln_Income | Logarithm of personal total income (RMB) | 4399 | 8.835 | 3.627 | 0 | 16.113 |
| Whether having pension | Yes=1, No=0 | 4648 | 0.750 | 0.433 | 0 | 1 |
| Whether having medical insurance | Yes=1, No=0 | 4652 | 0.936 | 0.245 | 0 | 1 |
| Family Characteristics |  |  |  |  |  |  |
| Whether married | Yes=1, No=0 | 4655 | 0.900 | 0.300 | 0 | 1 |
| Number of children | Number of children in the family | 4647 | 1.532 | 0.882 | 0 | 20 |
| Family size | Number of members in the family | 4650 | 2.954 | 1.294 | 1 | 30 |
| Number of houses | Number of houses in the family | 4616 | 1.108 | 0.637 | 0 | 12 |
| Year dummies |  |  |  |  |  |  |
| Province dummies |  |  |  |  |  |  |

**Supplementary Table 5.** Summary statistics of people in the age of 50-60

| **Variable** | **Description** | **Obs.** | **Mean** | **Std. Dev.** | **Min** | **Max** |
| --- | --- | --- | --- | --- | --- | --- |
| Explanatory Variables |  |  |  |  |  |  |
| Happiness | 1-5 levels | 4987 | 3.788 | 0.901 | 1 | 5 |
| Dependent Variable |  |  |  |  |  |  |
| Perceived Depression | 1-5 levels | 4987 | 2.229 | 1.031 | 1 | 5 |
| Control Variables |  |  |  |  |  |  |
| Demographic Characteristics |  |  |  |  |  |  |
| Whether female | Yes=1, No=0 | 4987 | 0.538 | 0.499 | 0 | 1 |
| Age | Age | 4987 | 55.437 | 2.857 | 51 | 60 |
| Age_squared | Squared term of age | 4987 | 3081.373 | 318.157 | 2601 | 3600 |
| Human Capital Characteristics |  |  |  |  |  |  |
| Education level | 1-13 levels | 4984 | 4.436 | 2.431 | 1 | 13 |
| Whether migrants | Yes=1, No=0 | 4983 | 0.091 | 0.288 | 0 | 1 |
| Social Characteristics |  |  |  |  |  |  |
| Whether Hukou in urban | Yes=1, No=0 | 4977 | 0.355 | 0.479 | 0 | 1 |
| Whether ethnic minorities | Yes=1, No=0 | 4987 | 0.074 | 0.261 | 0 | 1 |
| Whether religious believer | Yes=1, No=0 | 4987 | 0.114 | 0.317 | 0 | 1 |
| Whether CPC member | Yes=1, No=0 | 4983 | 0.094 | 0.292 | 0 | 1 |
| Social contacts frequency | 1-5 levels | 4986 | 2.735 | 1.085 | 1 | 5 |
| Working Characteristics |  |  |  |  |  |  |
| ln_Income | Logarithm of personal total income (RMB) | 4756 | 8.138 | 3.850 | 0 | 16.111 |
| Whether having pension | Yes=1, No=0 | 4981 | 0.799 | 0.401 | 0 | 1 |
| Whether having medical insurance | Yes=1, No=0 | 4981 | 0.941 | 0.235 | 0 | 1 |
| Family Characteristics |  |  |  |  |  |  |
| Whether married | Yes=1, No=0 | 4987 | 0.869 | 0.337 | 0 | 1 |
| Number of children | Number of children in the family | 4981 | 1.709 | 1.052 | 0 | 32 |
| Family size | Number of members in the family | 4983 | 2.755 | 1.486 | 1 | 23 |
| Number of houses | Number of houses in the family | 4947 | 1.110 | 0.563 | 0 | 10 |
| Year dummies |  |  |  |  |  |  |
| Province dummies |  |  |  |  |  |  |

**Supplementary Table 6.** Summary statistics of people in the age of 60-70

| **Variable** | **Description** | **Obs.** | **Mean** | **Std. Dev.** | **Min** | **Max** |
| --- | --- | --- | --- | --- | --- | --- |
| Explanatory Variables |  |  |  |  |  |  |
| Happiness | 1-5 levels | 4970 | 3.891 | 0.844 | 1 | 5 |
| Dependent Variable |  |  |  |  |  |  |
| Perceived Depression | 1-5 levels | 4970 | 2.225 | 1.008 | 1 | 5 |
| Control Variables |  |  |  |  |  |  |
| Demographic Characteristics |  |  |  |  |  |  |
| Whether female | Yes=1, No=0 | 4970 | 0.519 | 0.500 | 0 | 1 |
| Age | Age | 4970 | 65.258 | 2.769 | 61 | 70 |
| Age_squared | Squared term of age | 4970 | 4266.238 | 362.508 | 3721 | 4900 |
| Human Capital Characteristics |  |  |  |  |  |  |
| Education level | 1-13 levels | 4967 | 3.661 | 2.323 | 1 | 13 |
| Whether migrants | Yes=1, No=0 | 4966 | 0.076 | 0.265 | 0 | 1 |
| Social Characteristics |  |  |  |  |  |  |
| Whether Hukou in urban | Yes=1, No=0 | 4952 | 0.375 | 0.484 | 0 | 1 |
| Whether ethnic minorities | Yes=1, No=0 | 4970 | 0.058 | 0.233 | 0 | 1 |
| Whether religious believer | Yes=1, No=0 | 4970 | 0.114 | 0.318 | 0 | 1 |
| Whether CPC member | Yes=1, No=0 | 4968 | 0.135 | 0.341 | 0 | 1 |
| Social contacts frequency | 1-5 levels | 4969 | 2.716 | 1.136 | 1 | 5 |
| Working Characteristics |  |  |  |  |  |  |
| ln_Income | Logarithm of personal total income (RMB) | 4774 | 8.176 | 3.452 | 0 | 16.013 |
| Whether having pension | Yes=1, No=0 | 4967 | 0.837 | 0.369 | 0 | 1 |
| Whether having medical insurance | Yes=1, No=0 | 4968 | 0.936 | 0.244 | 0 | 1 |
| Family Characteristics |  |  |  |  |  |  |
| Whether married | Yes=1, No=0 | 4970 | 0.795 | 0.404 | 0 | 1 |
| Number of children | Number of children in the family | 4962 | 2.033 | 1.193 | 0 | 22 |
| Family size | Number of members in the family | 4967 | 2.594 | 1.484 | 1 | 23 |
| Number of houses | Number of houses in the family | 4937 | 1.109 | 0.603 | 0 | 12 |
| Year dummies |  |  |  |  |  |  |
| Province dummies |  |  |  |  |  |  |

**Supplementary Table 7.** Summary statistics of people above the age 70

| **Variable** | **Description** | **Obs.** | **Mean** | **Std. Dev.** | **Min** | **Max** |
| --- | --- | --- | --- | --- | --- | --- |
| Explanatory Variables |  |  |  |  |  |  |
| Happiness | 1-5 levels | 3415 | 4.014 | 0.823 | 1 | 5 |
| Dependent Variable |  |  |  |  |  |  |
| Perceived Depression | 1-5 levels | 3415 | 2.206 | 0.995 | 1 | 5 |
| Control Variables |  |  |  |  |  |  |
| Demographic Characteristics |  |  |  |  |  |  |
| Whether female | Yes=1, No=0 | 3415 | 0.526 | 0.499 | 0 | 1 |
| Age | Age | 3415 | 77.526 | 5.336 | 71 | 118 |
| Age_squared | Squared term of age | 3415 | 6038.780 | 856.114 | 6041 | 13924 |
| Human Capital Characteristics |  |  |  |  |  |  |
| Education level | 1-13 levels | 3414 | 3.565 | 2.739 | 1 | 13 |
| Whether migrants | Yes=1, No=0 | 3410 | 0.069 | 0.254 | 0 | 1 |
| Social Characteristics |  |  |  |  |  |  |
| Whether Hukou in urban | Yes=1, No=0 | 3408 | 0.415 | 0.493 | 0 | 1 |
| Whether ethnic minorities | Yes=1, No=0 | 3415 | 0.074 | 0.261 | 0 | 1 |
| Whether religious believer | Yes=1, No=0 | 3415 | 0.134 | 0.340 | 0 | 1 |
| Whether CPC member | Yes=1, No=0 | 3413 | 0.186 | 0.389 | 0 | 1 |
| Social contacts frequency | 1-5 levels | 3414 | 2.650 | 1.161 | 1 | 5 |
| Working Characteristics |  |  |  |  |  |  |
| ln_Income | Logarithm of personal total income (RMB) | 3273 | 7.975 | 3.689 | 0 | 16.117 |
| Whether having pension | Yes=1, No=0 | 3404 | 0.808 | 0.394 | 0 | 1 |
| Whether having medical insurance | Yes=1, No=0 | 3404 | 0.920 | 0.272 | 0 | 1 |
| Family Characteristics |  |  |  |  |  |  |
| Whether married | Yes=1, No=0 | 3415 | 0.558 | 0.497 | 0 | 1 |
| Number of children | Number of children in the family | 3404 | 3.040 | 1.665 | 0 | 24 |
| Family size | Number of members in the family | 3413 | 2.148 | 1.240 | 1 | 15 |
| Number of houses | Number of houses in the family | 3371 | 1.043 | 0.608 | 0 | 11 |
| Year dummies |  |  |  |  |  |  |
| Province dummies |  |  |  |  |  |  |

**Supplementary Table 8.** Cohort variations

| **Model** | **(1) Oprobit** | **(2) Oprobit** | **(3) Oprobit** | **(4) Oprobit** | **(5) Oprobit** | **(6) Oprobit** |
| --- | --- | --- | --- | --- | --- | --- |
| **Age Sample** | **20-30** | **30-40** | **40-50** | **50-60** | **60-70** | **>70** |
| **Variable** | **Perceived**  **depression** | **Perceived**  **depression** | **Perceived**  **depression** | **Perceived**  **depression** | **Perceived**  **depression** | **Perceived**  **depression** |
| Happiness | -0.363^***^  (0.033) | -0.352^***^  (0.029) | -0.413^***^  (0.023) | -0.383^***^  (0.021) | -0.391^***^  (0.021) | -0.385^***^  (0.025) |
| Whether female | 0.189^***^  (0.045) | 0.072^*^  (0.040) | 0.147^***^  (0.035) | 0.120^***^  (0.034) | 0.156^***^  (0.034) | 0.127^***^  (0.042) |
| Age | -0.263  (0.161) | 0.373^**^  (0.179) | 0.065  (0.206) | 0.243  (0.240) | 0.206  (0.295) | -0.007  (0.069) |
| Age_squared | 0.005^*^  (0.003) | -0.005^**^  (0.003) | -0.001  (0.002) | -0.002  (0.002) | -0.002  (0.002) | 0.000  (0.000) |
| Education level | 0.007  (0.008) | 0.003  (0.008) | -0.008  (0.008) | -0.012  (0.008) | -0.034^***^  (0.009) | -0.025^***^  (0.009) |
| Whether migrants | 0.064  (0.055) | 0.102^*^  (0.054) | -0.128^**^  (0.057) | 0.003  (0.062) | -0.181^***^  (0.069) | 0.005  (0.080) |
| Whether Hukou in urban | 0.040  (0.051) | -0.015  (0.048) | -0.019  (0.043) | -0.096^**^  (0.043) | -0.166^***^  (0.044) | -0.146^***^  (0.053) |
| Whether ethnic minorities | 0.060  (0.092) | -0.049  (0.087) | -0.010  (0.074) | -0.041  (0.077) | -0.109  (0.082) | -0.099  (0.088) |
| Whether religious believer | 0.241^***^  (0.088) | 0.090  (0.075) | 0.005  (0.061) | -0.030  (0.054) | 0.111^**^  (0.055) | 0.057  (0.060) |
| Whether CPC member | -0.062  (0.083) | 0.003  (0.067) | -0.149^**^  (0.070) | -0.105^*^  (0.062) | -0.047  (0.055) | -0.055  (0.056) |
| Social contacts frequency | -0.026  (0.024) | -0.021  (0.021) | 0.001  (0.018) | -0.036^**^  (0.016) | -0.017  (0.015) | -0.068^***^  (0.017) |
| ln_Income | 0.001  (0.005) | -0.012^**^  (0.006) | -0.015^***^  (0.005) | -0.015^***^  (0.005) | -0.021^***^  (0.005) | -0.011^*^  (0.006) |
| Whether having pension | -0.066  (0.050) | -0.058  (0.047) | -0.075^*^  (0.042) | -0.061  (0.043) | 0.007  (0.046) | -0.006  (0.053) |
| Whether having medical insurance | 0.155^**^  (0.077) | 0.140  (0.091) | 0.144^*^  (0.078) | 0.151^**^  (0.075) | -0.025  (0.071) | 0.066  (0.075) |
| Whether married | -0.066  (0.057) | -0.055  (0.064) | -0.052  (0.059) | -0.122^**^  (0.048) | -0.034  (0.040) | -0.060  (0.043) |
| Number of children | -0.009  (0.031) | 0.002  (0.025) | -0.005  (0.019) | 0.053^**^  (0.021) | 0.021  (0.014) | 0.014  (0.013) |
| Family size | 0.002  (0.014) | -0.014  (0.016) | 0.004  (0.014) | 0.011  (0.011) | -0.020^*^  (0.011) | -0.026  (0.017) |
| Number of houses | 0.046  (0.031) | 0.038  (0.031) | 0.011  (0.028) | -0.011  (0.031) | -0.067^**^  (0.030) | -0.062^*^  (0.033) |
| Year dummies | Yes | Yes | Yes | Yes | Yes | Yes |
| Province dummies | Yes | Yes | Yes | Yes | Yes | Yes |
| Observations | 2685 | 3384 | 4342 | 4698 | 4716 | 3205 |
| Adjusted R^2^ | 0.041 | 0.043 | 0.065 | 0.075 | 0.080 | 0.073 |

**Supplementary Table 9.** Robustness tests: using different subjective well-being indicators

| **Model** | **(1) Oprobit** | **(2) Oprobit** | **(3) Oprobit** | **(4) Oprobit** | **(5) Oprobit** | **(6) Oprobit** | **(7) Oprobit** |
| --- | --- | --- | --- | --- | --- | --- | --- |
| **Variable** | **Perceived**  **depression** | **Perceived**  **depression** | **Perceived**  **depression** | **Perceived**  **depression** | **Perceived**  **depression** | **Perceived**  **depression** | **Perceived**  **depression** |
| Evaluative well-being | -0.370^***^  (0.024) |  |  |  |  |  |  |
| Experienced well-being_1 |  | -0.090^***^  (0.019) |  |  |  |  |  |
| Experienced well-being_2 |  |  | -0.181^***^  (0.019) |  |  |  |  |
| Experienced well-being_3 |  |  |  | -0.122^***^  (0.020) |  |  |  |
| Eudaimonic well-being_1 |  |  |  |  | -0.101^***^  (0.020) |  |  |
| Eudaimonic well-being_2 |  |  |  |  |  | -0.104^***^  (0.017) |  |
| Eudaimonic well-being_3 |  |  |  |  |  |  | -0.087^***^  (0.017) |
| Whether female | 0.125^***^  (0.037) | 0.062^*^  (0.037) | 0.105^***^  (0.037) | 0.072^*^  (0.037) | 0.064^*^  (0.037) | 0.071^*^  (0.037) | 0.066^*^  (0.037) |
| Age | 0.029^***^  (0.007) | 0.036^***^  (0.007) | 0.034^***^  (0.007) | 0.036^***^  (0.007) | 0.032^***^  (0.007) | 0.036^***^  (0.007) | 0.032^***^  (0.007) |
| Age_squared | -0.000^***^  (0.000) | -0.000^***^  (0.000) | -0.000^***^  (0.000) | -0.000^***^  (0.000) | -0.000^***^  (0.000) | -0.000^***^  (0.000) | -0.000^***^  (0.000) |
| Education level | -0.006  (0.008) | -0.012  (0.008) | -0.012  (0.008) | -0.010  (0.008) | -0.011  (0.008) | -0.011  (0.008) | -0.012  (0.008) |
| Whether migrants | 0.103^*^  (0.060) | 0.114^*^  (0.060) | 0.111^*^  (0.060) | 0.110^*^  (0.060) | 0.127^**^  (0.060) | 0.131^**^  (0.060) | 0.139^**^  (0.060) |
| Whether Hukou in urban | -0.100^**^  (0.045) | -0.125^***^  (0.045) | -0.103^**^  (0.045) | -0.129^***^  (0.046) | -0.108^**^  (0.045) | -0.113^**^  (0.046) | -0.107^**^  (0.046) |
| Whether ethnic minorities | 0.043  (0.082) | -0.006  (0.082) | -0.019  (0.084) | -0.017  (0.082) | -0.014  (0.083) | 0.009  (0.081) | 0.001  (0.081) |
| Whether religious believer | 0.027  (0.065) | 0.042  (0.067) | 0.053  (0.066) | 0.038  (0.067) | 0.045  (0.068) | 0.057  (0.067) | 0.067  (0.067) |
| Whether CPC member | -0.136^**^  (0.062) | -0.177^***^  (0.062) | -0.153^**^  (0.062) | -0.157^**^  (0.062) | -0.164^***^  (0.063) | -0.163^***^  (0.063) | -0.168^***^  (0.063) |
| Social contacts frequency | -0.025  (0.018) | -0.041^**^  (0.018) | -0.031^*^  (0.018) | -0.038^**^  (0.018) | -0.038^**^  (0.018) | -0.035^*^  (0.018) | -0.033^*^  (0.018) |
| ln_Income | -0.015^***^  (0.005) | -0.019^***^  (0.005) | -0.019^***^  (0.005) | -0.019^***^  (0.005) | -0.018^***^  (0.005) | -0.017^***^  (0.005) | -0.018^***^  (0.005) |
| Whether having pension | -0.019  (0.045) | -0.026  (0.046) | -0.024  (0.046) | -0.041  (0.046) | -0.031  (0.046) | -0.043  (0.046) | -0.028  (0.046) |
| Whether having medical insurance | 0.159^**^  (0.077) | 0.145^*^  (0.079) | 0.142^*^  (0.079) | 0.145^*^  (0.079) | 0.121  (0.080) | 0.148^*^  (0.080) | 0.146^*^  (0.079) |
| Whether married | -0.184^***^  (0.048) | -0.241^***^  (0.048) | -0.217^***^  (0.048) | -0.231^***^  (0.049) | -0.228^***^  (0.049) | -0.225^***^  (0.048) | -0.227^***^  (0.049) |
| Number of children | 0.036^**^  (0.018) | 0.033^*^  (0.018) | 0.034^*^  (0.019) | 0.034^*^  (0.018) | 0.032^*^  (0.019) | 0.032^*^  (0.018) | 0.030^*^  (0.018) |
| Family size | 0.018  (0.014) | 0.007  (0.014) | 0.012  (0.014) | 0.009  (0.014) | 0.009  (0.014) | 0.010  (0.014) | 0.006  (0.014) |
| Number of houses | -0.024  (0.030) | -0.060^**^  (0.030) | -0.036  (0.030) | -0.061^**^  (0.030) | -0.060^**^  (0.030) | -0.054^*^  (0.030) | -0.063^**^  (0.030) |
| Year dummies | Yes | Yes | Yes | Yes | Yes | Yes | Yes |
| Province dummies | Yes | Yes | Yes | Yes | Yes | Yes | Yes |
| Observations | 3837 | 3761 | 3777 | 3762 | 3716 | 3768 | 3716 |
| Pseudo R^2^ | 0.064 | 0.040 | 0.047 | 0.041 | 0.040 | 0.042 | 0.041 |

Note: The variable of Evaluative well-being comes from the respondents’ degree of agreement on “In general, how are you satisfied with your life?”. Experienced well-being_1, Experienced well-being_2 and Experienced well-being_3 come from the respondents’ degree of agreement on “I am content with my life compared with others around me”, “I am satisfied with my family’s income level” and “I feel confident about the development of society” respectively. Eudaimonic well-being_1, Eudaimonic well-being_2 and Eudaimonic well-being_3 come from the respondents’ degree of agreement on “Most of the goals I set for my life give me encouragement to live better”, “I feel I have purpose and hope in my life” and “I am very clear about the meaning of my life” respectively.

**Supplementary Table 10.** Robustness tests: using another depressive disorders indicator

| **Model** | **(1) Probit** | **(2) Probit** | **(3) Probit** | **(4) Probit** | **(5) Probit** | **(6) Probit** | **(7) Probit** |
| --- | --- | --- | --- | --- | --- | --- | --- |
| **Variable** | **Whe_depression** | **Whe_depression** | **Whe_depression** | **Whe_depression** | **Whe_depression** | **Whe_depression** | **Whe_depression** |
| Happiness | -0.409^***^  (0.010) | -0.416^***^  (0.010) | -0.402^***^  (0.010) | -0.396^***^  (0.011) | -0.391^***^  (0.011) | -0.386^***^  (0.011) | -0.386^***^  (0.011) |
| Whether female |  | 0.186^***^  (0.017) | 0.160^***^  (0.017) | 0.163^***^  (0.017) | 0.144^***^  (0.018) | 0.142^***^  (0.018) | 0.156^***^  (0.018) |
| Age |  | 0.008^***^  (0.003) | -0.001  (0.003) | 0.002  (0.003) | 0.006^**^  (0.003) | 0.014^***^  (0.003) | 0.013^***^  (0.004) |
| Age_squared |  | -0.000  (0.000) | 0.000  (0.000) | 0.000  (0.000) | -0.000  (0.000) | -0.000^***^  (0.000) | -0.000^***^  (0.000) |
| Education level |  |  | -0.037^***^  (0.003) | -0.015^***^  (0.004) | -0.012^***^  (0.004) | -0.010^**^  (0.004) | -0.006  (0.004) |
| Whether migrants |  |  | -0.031  (0.025) | -0.042^*^  (0.026) | -0.031  (0.027) | -0.038  (0.027) | 0.026  (0.029) |
| Whether Hukou in urban |  |  |  | -0.196^***^  (0.020) | -0.171^***^  (0.021) | -0.150^***^  (0.022) | -0.095^***^  (0.023) |
| Whether ethnic minorities |  |  |  | 0.155^***^  (0.032) | 0.142^***^  (0.033) | 0.131^***^  (0.034) | -0.004  (0.040) |
| Whether religious believer |  |  |  | 0.075^***^  (0.028) | 0.076^***^  (0.028) | 0.064^**^  (0.029) | 0.076^**^  (0.030) |
| Whether CPC member |  |  |  | -0.105^***^  (0.030) | -0.085^***^  (0.031) | -0.072^**^  (0.031) | -0.089^***^  (0.031) |
| Social contacts frequency |  |  |  | -0.039^***^  (0.008) | -0.038^***^  (0.008) | -0.041^***^  (0.008) | -0.035^***^  (0.008) |
| ln_Income |  |  |  |  | -0.020^***^  (0.002) | -0.019^***^  (0.002) | -0.016^***^  (0.002) |
| Whether having pension |  |  |  |  | -0.016  (0.022) | -0.015  (0.022) | -0.010  (0.023) |
| Whether having medical insurance |  |  |  |  | 0.071^**^  (0.036) | 0.077^**^  (0.036) | 0.066^*^  (0.036) |
| Whether married |  |  |  |  |  | -0.123^***^  (0.024) | -0.113^***^  (0.024) |
| Number of children |  |  |  |  |  | 0.056^***^  (0.009) | 0.036^***^  (0.009) |
| Family size |  |  |  |  |  | -0.003  (0.007) | -0.005  (0.007) |
| Number of houses |  |  |  |  |  | 0.004  (0.014) | 0.016  (0.014) |
| Year dummies | No | No | No | No | No | No | Yes |
| Province dummies | No | No | No | No | No | No | Yes |
| Constant | 1.164^***^  (0.040) | 0.773^***^  (0.082) | 1.219^***^  (0.089) | 1.119^***^  (0.093) | 1.078^***^  (0.101) | 0.931^***^  (0.108) | 0.534^***^  (0.118) |
| Observations | 25278 | 25278 | 25195 | 25111 | 23749 | 23515 | 23515 |
| Pseudo R^2^ | 0.053 | 0.060 | 0.064 | 0.070 | 0.071 | 0.073 | 0.086 |

**Supplementary Table 11.** Robustness tests: using Ordered-Logit model

| **Model** | **(1) Ologit** | **(2) Ologit** | **(3) Ologit** | **(4) Ologit** | **(5) Ologit** | **(6) Ologit** | **(7) Ologit** |
| --- | --- | --- | --- | --- | --- | --- | --- |
| **Variable** | **Perceived**  **depression** | **Perceived**  **depression** | **Perceived**  **depression** | **Perceived**  **depression** | **Perceived**  **depression** | **Perceived**  **depression** | **Perceived**  **depression** |
| Happiness | -0.748^***^  (0.016) | -0.760^***^  (0.016) | -0.732^***^  (0.017) | -0.722^***^  (0.017) | -0.715^***^  (0.017) | -0.704^***^  (0.017) | -0.697^***^  (0.018) |
| Whether female |  | 0.279^***^  (0.023) | 0.236^***^  (0.023) | 0.237^***^  (0.024) | 0.208^***^  (0.025) | 0.203^***^  (0.025) | 0.237^***^  (0.025) |
| Age |  | 0.007^**^  (0.004) | -0.010^**^  (0.004) | -0.003  (0.004) | 0.005  (0.004) | 0.015^***^  (0.005) | 0.015^***^  (0.005) |
| Age_squared |  | 0.000  (0.000) | 0.000^***^  (0.000) | 0.000^**^  (0.000) | 0.000  (0.000) | -0.000^***^  (0.000) | -0.000^*^  (0.000) |
| Education level |  |  | -0.065^***^  (0.004) | -0.026^***^  (0.005) | -0.019^***^  (0.005) | -0.017^***^  (0.005) | -0.004  (0.005) |
| Whether migrants |  |  | -0.175^***^  (0.036) | -0.197^***^  (0.037) | -0.173^***^  (0.038) | -0.187^***^  (0.039) | -0.003  (0.041) |
| Whether Hukou in urban |  |  |  | -0.359^***^  (0.027) | -0.324^***^  (0.029) | -0.291^***^  (0.029) | -0.163^***^  (0.031) |
| Whether ethnic minorities |  |  |  | 0.202^***^  (0.047) | 0.192^***^  (0.048) | 0.176^***^  (0.048) | -0.071  (0.057) |
| Whether religious believer |  |  |  | 0.081^**^  (0.040) | 0.069^*^  (0.042) | 0.057  (0.042) | 0.104^**^  (0.044) |
| Whether CPC member |  |  |  | -0.222^***^  (0.042) | -0.193^***^  (0.043) | -0.171^***^  (0.044) | -0.195^***^  (0.044) |
| Social contacts frequency |  |  |  | -0.040^***^  (0.012) | -0.038^***^  (0.012) | -0.040^***^  (0.012) | -0.037^***^  (0.013) |
| ln_Income |  |  |  |  | -0.030^***^  (0.003) | -0.029^***^  (0.003) | -0.021^***^  (0.004) |
| Whether having pension |  |  |  |  | -0.058^*^  (0.030) | -0.058^*^  (0.030) | -0.021  (0.031) |
| Whether having medical insurance |  |  |  |  | 0.169^***^  (0.051) | 0.189^***^  (0.052) | 0.162^***^  (0.052) |
| Whether married |  |  |  |  |  | -0.178^***^  (0.033) | -0.163^***^  (0.033) |
| Number of children |  |  |  |  |  | 0.087^***^  (0.013) | 0.053^***^  (0.012) |
| Family size |  |  |  |  |  | -0.015  (0.009) | -0.017^*^  (0.010) |
| Number of houses |  |  |  |  |  | -0.022  (0.021) | 0.004  (0.021) |
| Year dummies | No | No | No | No | No | No | Yes |
| Province dummies | No | No | No | No | No | No | Yes |
| Observations | 25278 | 25278 | 25195 | 25111 | 23749 | 23515 | 23515 |
| Pseudo R^2^ | 0.039 | 0.043 | 0.047 | 0.051 | 0.052 | 0.053 | 0.061 |

**Supplementary Table 12.** Robustness tests: using Ordinary Least Squares model

| **Model** | **(1) OLS** | **(2) OLS** | **(3) OLS** | **(4) OLS** | **(5) OLS** | **(6) OLS** | **(7) OLS** |
| --- | --- | --- | --- | --- | --- | --- | --- |
| **Variable** | **Perceived**  **depression** | **Perceived**  **depression** | **Perceived**  **depression** | **Perceived**  **depression** | **Perceived**  **depression** | **Perceived**  **depression** | **Perceived**  **depression** |
| Happiness | -0.369^***^  (0.008) | -0.373^***^  (0.008) | -0.358^***^  (0.008) | -0.351^***^  (0.008) | -0.346^***^  (0.008) | -0.341^***^  (0.008) | -0.335^***^  (0.008) |
| Whether female |  | 0.142^***^  (0.012) | 0.118^***^  (0.012) | 0.119^***^  (0.012) | 0.102^***^  (0.012) | 0.100^***^  (0.012) | 0.114^***^  (0.012) |
| Age |  | 0.005^***^  (0.002) | -0.003^*^  (0.002) | -0.000  (0.002) | 0.004^*^  (0.002) | 0.009^***^  (0.002) | 0.009^***^  (0.002) |
| Age_squared |  | -0.000  (0.000) | 0.000^**^  (0.000) | 0.000  (0.000) | -0.000  (0.000) | -0.000^***^  (0.000) | -0.000^**^  (0.000) |
| Education level |  |  | -0.032^***^  (0.002) | -0.013^***^  (0.002) | -0.009^***^  (0.003) | -0.008^***^  (0.003) | -0.003  (0.003) |
| Whether migrants |  |  | -0.075^***^  (0.017) | -0.086^***^  (0.018) | -0.073^***^  (0.018) | -0.079^***^  (0.018) | 0.001  (0.019) |
| Whether Hukou in urban |  |  |  | -0.176^***^  (0.013) | -0.157^***^  (0.014) | -0.140^***^  (0.014) | -0.082^***^  (0.015) |
| Whether ethnic minorities |  |  |  | 0.106^***^  (0.024) | 0.097^***^  (0.024) | 0.088^***^  (0.024) | -0.030  (0.028) |
| Whether religious believer |  |  |  | 0.051^**^  (0.020) | 0.046^**^  (0.021) | 0.040^*^  (0.021) | 0.056^***^  (0.021) |
| Whether CPC member |  |  |  | -0.092^***^  (0.020) | -0.077^***^  (0.020) | -0.065^***^  (0.020) | -0.076^***^  (0.020) |
| Social contacts frequency |  |  |  | -0.026^***^  (0.006) | -0.025^***^  (0.006) | -0.026^***^  (0.006) | -0.023^***^  (0.006) |
| ln_Income |  |  |  |  | -0.016^***^  (0.002) | -0.015^***^  (0.002) | -0.012^***^  (0.002) |
| Whether having pension |  |  |  |  | -0.025  (0.015) | -0.024  (0.015) | -0.011  (0.016) |
| Whether having medical insurance |  |  |  |  | 0.066^**^  (0.026) | 0.074^***^  (0.026) | 0.061^**^  (0.026) |
| Whether married |  |  |  |  |  | -0.091^***^  (0.016) | -0.083^***^  (0.016) |
| Number of children |  |  |  |  |  | 0.044^***^  (0.006) | 0.027^***^  (0.006) |
| Family size |  |  |  |  |  | -0.008^*^  (0.005) | -0.009^**^  (0.005) |
| Number of houses |  |  |  |  |  | -0.006  (0.010) | 0.005  (0.010) |
| Year dummies | No | No | No | No | No | No | Yes |
| Province dummies | No | No | No | No | No | No | Yes |
| Constant | 3.594^***^  (0.031) | 3.296^***^  (0.056) | 3.711^***^  (0.060) | 3.593^***^  (0.063) | 3.546^***^  (0.069) | 3.451^***^  (0.073) | 3.037^***^  (0.079) |
| Observations | 25278 | 25278 | 25195 | 25111 | 23749 | 23515 | 23515 |
| Adjusted R^2^ | 0.098 | 0.109 | 0.119 | 0.127 | 0.129 | 0.132 | 0.149 |

**Supplementary Table 13.** Robustness tests: using panelized machine learning methods

| **Model** | **(1)** | **(2)** | **(3)** | **(4)** | **(5)** | **(6)** |
| --- | --- | --- | --- | --- | --- | --- |
|  | **Lasso**  **(10-fold CV)** | **Lasso**  **(20-fold CV)** | **Ridge**  **(10-fold CV)** | **Ridge**  **(20-fold CV)** | **Elastic Net**  **(10-fold CV)** | **Elastic Net**  **(20-fold CV)** |
| **Variable** | **Perceived**  **depression** | **Perceived**  **depression** | **Perceived**  **depression** | **Perceived**  **depression** | **Perceived**  **depression** | **Perceived**  **depression** |
| Happiness | -0.3350 | -0.3351 | -0.3264 | -0.3264 | -0.3350 | -0.3348 |
| No. of nonzero coefficients | 45 | 45 | 48 | 48 | 45 | 47 |
| $\lambda$ | 0.0003 | 0.0003 | 0.0304 | 0.0304 | 0.0003 | 0.0012 |
| Out-of-sample R^2^ | 0.1462 | 0.1464 | 0.1461 | 0.1463 | 0.1462 | 0.1464 |
| CV mean prediction error | 0.8195 | 0.8193 | 0.8197 | 0.8195 | 0.8195 | 0.8193 |
| $\alpha$ |  |  |  |  | 1 | 0.2 |
| Observations | 23515 | 23515 | 23515 | 23515 | 23515 | 23515 |

**Supplementary Table 14.**Dealing with endogeneity: instrumental variable regressions

| **Model** | **(1)** | **(2)** | **(3)** | **(4)** | **(5)** |
| --- | --- | --- | --- | --- | --- |
|  | **First Stage** | **2SLS**  **Second Stage** | **LIML**  **Second Stage** | **GMM**  **Second Stage** | **IGMM**  **Second Stage** |
| **Variable** | **Happiness** | **Perceived**  **depression** | **Perceived**  **depression** | **Perceived**  **depression** | **Perceived**  **depression** |
| Happiness |  | -1.341^***^  (0.461) | -1.341^***^  (0.461) | -1.341^***^  (0.461) | -1.341^***^  (0.461) |
| Non-routine tasks | 0.091^***^  (0.027) |  |  |  |  |
| Whether female | 0.043^***^  (0.015) | 0.187^***^  (0.031) | 0.187^***^  (0.031) | 0.187^***^  (0.031) | 0.187^***^  (0.031) |
| Age | -0.036^***^  (0.004) | -0.033^*^  (0.017) | -0.033^*^  (0.017) | -0.033^*^  (0.017) | -0.033^*^  (0.017) |
| Age_squared | 0.000^***^  (0.000) | 0.000^**^  (0.000) | 0.000^**^  (0.000) | 0.000^**^  (0.000) | 0.000^**^  (0.000) |
| Education level | 0.020^***^  (0.003) | 0.020^*^  (0.010) | 0.020^*^  (0.010) | 0.020^*^  (0.010) | 0.020^*^  (0.010) |
| Whether migrants | -0.027  (0.022) | 0.020  (0.036) | 0.020  (0.036) | 0.020  (0.036) | 0.020  (0.036) |
| Whether Hukou in urban | 0.008  (0.019) | -0.025  (0.030) | -0.025  (0.030) | -0.025  (0.030) | -0.025  (0.030) |
| Whether ethnic minorities | 0.118^***^  (0.033) | 0.035  (0.074) | 0.035  (0.074) | 0.035  (0.074) | 0.035  (0.074) |
| Whether religious believer | 0.016  (0.028) | 0.090^**^  (0.043) | 0.090^**^  (0.043) | 0.090^**^  (0.043) | 0.090^**^  (0.043) |
| Whether CPC member | 0.120^***^  (0.023) | 0.024  (0.066) | 0.024  (0.066) | 0.024  (0.066) | 0.024  (0.066) |
| Social contacts frequency | 0.059^***^  (0.008) | 0.046  (0.030) | 0.046  (0.030) | 0.046  (0.030) | 0.046  (0.030) |
| ln_Income | 0.018^***^  (0.003) | 0.006  (0.010) | 0.006  (0.010) | 0.006  (0.010) | 0.006  (0.010) |
| Whether having pension | 0.050^**^  (0.020) | 0.034  (0.038) | 0.034  (0.038) | 0.034  (0.038) | 0.034  (0.038) |
| Whether having medical insurance | 0.017  (0.035) | 0.104^**^  (0.051) | 0.104^**^  (0.051) | 0.104^**^  (0.051) | 0.104^**^  (0.051) |
| Whether married | 0.262^***^  (0.024) | 0.174  (0.126) | 0.174  (0.126) | 0.174  (0.126) | 0.174  (0.126) |
| Number of children | 0.009  (0.009) | 0.026^*^  (0.014) | 0.026^*^  (0.014) | 0.026^*^  (0.014) | 0.026^*^  (0.014) |
| Family size | 0.020^***^  (0.006) | 0.025^*^  (0.013) | 0.025^*^  (0.013) | 0.025^*^  (0.013) | 0.025^*^  (0.013) |
| Number of houses | 0.077^***^  (0.011) | 0.097^**^  (0.040) | 0.097^**^  (0.040) | 0.097^**^  (0.040) | 0.097^**^  (0.040) |
| Year dummies | Yes | Yes | Yes | Yes | Yes |
| Province dummies | Yes | Yes | Yes | Yes | Yes |
| Constant | 3.791^***^  (0.102) | 6.839^***^  (1.757) | 6.839^***^  (1.757) | 6.839^***^  (1.757) | 6.839^***^  (1.757) |
| Observations | 11837 | 11837 | 11837 | 11837 | 11837 |
| Adjusted R^2^ | 0.072 |  |  |  |  |

**Supplementary Figure 1.** Coefficient Paths in the Lasso/Elastic Net Model

**Supplementary Figure 2.** Coefficient Paths in the Ridge Model

**Supplementary Figure 3.** Placebo Test

**Supplementary Introduction to the Dataset**

This paper uses a nationally representative observational dataset, which is the Chinese General Social Survey (CGSS). CGSS is in the world General Social Survey family, carried out by the national Survey Research Center at Renmin University of China (NSRC), which organizes the Chinese Social Survey Network (CSSN) including 49 universities and provincial social science academies. CGSS aims to represent the adult population of Chinese Mainland (excluding Hong Kong, Macao, Taiwan and Tibet) over 18 years old, which is the universe of CGSS. According to the dataset in China Statistical Yearbook Survey (http://www.stats.gov.cn/tjsj./ndsj/), the total population of Chinese Mainland (excluding Hong Kong, Macao, Taiwan and Tibet) over 18 years old in 2017 and 2018 are 950766 and 949531 respectively. The sampling of CGSS is based on the multi-stage stratified design. The sampling stages are as follows: (1) PSUs are county-level units and there are 2762 PSUs in the sampling frame; (2) SSUs are community-level units (villages [cun] and neighborhood committees [ju wei hui]); (3) in selected SSU, 25 households (TSUs) are sampled with PPS method; (4) one eligible person aged 18-above is selected from each sampled household to serve as the representative. There are 43 Municipalities directly under the Chinese central government, provincial capital cities, and vice provincial cities in China. Comprehensive ranking by GDP, FDI and Education Level to these cities, the top 5 is Beijing, Shanghai, Tianjin, Guangzhou, and Shenzhen. CGSS treats these 5 cities as self-representative stratum. This stratum consists of 67 PSUs. The rest 2695 PSUs are comprehensively ranked with GDP per capital, urbanization rate, and population density and then are equally classified into 50 strata. Within each stratum, 2 PSUs will be selected with PPS method. In each selected PSU, 4 communities are sampled with PPS method. There are 80 communities in self-representative stratum and 400 communities in the rest 50 strata. The sample size for 2017 and 2018 are 12534 and 12744 respectively.

Above information is taken from http://cgss.ruc.edu.cn/English/Home.htm.
